# Complete Genome Sequences of *Thermus* Strains Isolated from Senami Hot Spring in Japan

**DOI:** 10.1128/mra.00354-22

**Published:** 2022-05-31

**Authors:** Kentaro Miyazaki

**Affiliations:** a Bioproduction Research Institute, National Institute of Advanced Industrial Science and Technology, Tsukuba, Ibaraki, Japan; b International Center for Biotechnology, Osaka University, Suita, Osaka, Japan; University of Delaware

## Abstract

Extremely thermophilic strains belonging to the genus *Thermus* were isolated from Senami Hot Spring in Japan. Here, I report the complete genome sequences of five Thermus thermophilus strains and one Thermus brockianus strain, which were obtained by combining Oxford Nanopore long-read and DNBSEQ or Illumina short-read sequencing data.

## ANNOUNCEMENT

Thermus aquaticus is the type species of the genus *Thermus*, which was first isolated from Yellowstone National Park in the United States in 1969 (1). At almost the same time, Thermus thermophilus was isolated from Mine Hot Spring in Japan (2, 3). Due to its high-temperature adaptation at around 70°C, *Thermus* has great biotechnological potential as a source of thermophilic enzymes, as exemplified by *Taq* DNA polymerase (4). Since the discovery of *T. aquaticus*, many *Thermus* strains have been isolated from thermal areas worldwide (5–11).

I collected boiling water samples at Senami Hot Spring (38.2139 N, 139.4438 E) in Japan. Samples were spread over *Thermus* medium (0.4% [wt/vol] yeast extract, 0.8% [wt/vol] peptone, 0.2% [wt/vol] NaCl) agar plates (1.6% [wt/vol]) containing 0.4 mM MgCl_2_ and 0.35 mM CaCl_2_ (*Thermus* MC medium) (10). After incubation at 75°C overnight, dozens of well-separated single colonies were isolated, and colony PCR was conducted to analyze the 16S rRNA gene using the Thermus_1F and Thermus_1521R primer sets (12). I selected five T. thermophilus strains (≥99.8% 16S rRNA identities to HB8^T^ [TTH_RS00710]) and one *T. brockianus* strain (99.9% 16S rRNA identity to YS38^T^ [NR_036983.1]) for whole-genome analysis.

To prepare genomic DNA, cells were aerobically grown in 5 mL of *Thermus* MC medium at 75°C for 24 h. Genomic DNA was purified using a blood and cell culture DNA midikit (Qiagen; catalog number 13323). For long-read sequencing, unsheared genomic DNA (1 μg) was treated with a short-read eliminator kit (Circulomics; catalog number SS-100-121-01) to remove fragments of <10 kbp, and a library was constructed using a ligation sequencing kit (Oxford Nanopore Technologies [ONT]; catalog number SQK-LSK109). Sequencing was performed with a GridION X5 system on a FLO-MIN106 R9.41 revD flow cell (ONT). Base calling was conducted using Guppy v.4.0.11. The raw data (Table 1) were filtered (*Q* >10; length, >1,000 bases) using NanoFilt v.2.7.1 (13). For short-read sequencing of SNM1-1, SNM3-3, and SNM1-7, a library was constructed using an MGIEasy FS PCR Free DNA library preparation set (MGI Tech Co., Ltd.; catalog number 1000013455) with a ~400- to 500-bp insert. Paired-end sequencing (2 × 150 bases) was then performed on a DNBSEQ-400 instrument (MGI). For SNM6-6, SNM7-6, and SNM4-1, the DNA prep kit (Illumina; catalog number 20018704) was used to generate paired-end libraries with approximately 350-bp inserts. Sequencing was performed using a MiSeq reagent kit v.2 (300 cycles) with 256-bp reads. These raw data were filtered (*Q* > 30; length, >20 bases) using fastp v.0.20.1 (14) (Table 1). Default parameters were used for all software. The trimmed long- and short-read data were assembled using Unicycler v.0.4.8 (15), and the assembly was polished using Pilon v.1.24 (16).

**TABLE 1:** Strains sequenced in this study

Strain	BioSample accession no.	Chromosome or plasmid	Short read			Long read				Length (bp)	GC content (%)	avg read depth (×)	No. of coding sequences	GenBank accession no.
			No. of paired end reads	Total length (Mb)	SRA accession no.	No. of reads	*N*_50_ (bases)	Total length (Mb)	SRA accession no.					
T. thermophilus SNM1-1	SAMD00442895		12,080,398	1,812	DRR346611	676,877	6,660	2,160	DRR346617					
		Chromosome								2,126,669	69.31	107	2,371	AP025596
		pTthSNM1-1b								102,381	69.50	866	116	AP025597
		pTthSNM1-1c								23,201	68.34	4,720	30	AP025598
		pTthSNM1-1d								10,007	70.83	5,654	8	AP025599
		pTthSNM1-1e								7,766	70.63	4,429	8	AP025600
		pTthSNM1-1f								4,719	71.92	12,980	4	AP025601
		pTthSNM1-1g								3,774	57.18	6,973	4	AP025602

T. thermophilus SNM3-3	SAMD00442896		5,407,329	811	DRR346612	1,066,704	3,727	2,995	DRR346618					
		Chromosome								1,971,781	69.34	676	2,113	AP025609
		pTthSNM3-3b								241,732	68.86	566	263	AP025610
		pTthSNM3-3c								153,987	68.68	966	171	AP025611
		pTthSNM3-3d								8,158	70.95	5,285	11	AP025612

T. thermophilus SNM6-6	SAMD00442897		963,253	226	DRR346613	1,459,467	3,609	2,897	DRR346619					
		Chromosome								1,945,718	69.41	136	2,087	AP025613
		pTthSNM6-6b								213,005	68.95	115	219	AP025614
		pTthSNM6-6c								79,572	68.25	163	96	AP025615
		pTthSNM6-6d								19,678	68.25	1,025	24	AP025616

T. thermophilus SNM7-6	SAMD00442898		989,094	227	DRR346614	1,608,666	1,807	2,619	DRR346620					
		Chromosome								1,848,270	69.53	123	1,978	AP025617
		pTthSNM7-6b								245,477	66.73	188	275	AP025618
		pTthSNM7-6c								198,886	69.51	115	218	AP025619
		pTthSNM7-6d								22,021	68.52	376	27	AP025620

T. thermophilus SNM1-7	SAMD00442899		4,871,052	731	DRR346615	1,096,885	13,982	3,978	DRR346621					
		Chromosome								1,870,712	69.44	381	2,022	AP025603
		pTthSNM1-7b								239,363	68.64	252	244	AP025604
		pTthSNM1-7c								132,169	68.74	365	149	AP025605
		pTthSNM1-7d								22,908	68.95	180	29	AP025606
		pTthSNM1-7e								15,350	67.95	2,223	18	AP025607
		pTthSNM1-7f								9,390	70.71	1,960	6	AP025608

*T. brockianus* SNM4-1	SAMD00442900		1,139,199	249	DRR346616	1,088,755	4,184	3,050	DRR346622					
		Chromosome								2,046,081	66.96	214	2,181	AP025593
		pTbrSNM4-1b								352,526	65.83	181	319	AP025594
		pTbrSNM4-1c								25,316	64.31	436	31	AP025595

All strains contained a single circular chromosome and multiple circular plasmids. Circularity was confirmed via Unicycler. Automatic annotation was conducted using DFAST v.1.2.15 (17); genomic features are summarized in Table 1.

### Data availability.

All six *Thermus* strains are associated with BioProject PRJDB12526. The BioSample accession numbers and accession numbers for genome sequences and raw sequencing data are available in Table 1.
